# Computer Vision-Assisted Measurement of Ephemeral Gully Morphology Using a Portable Pin-Artboard Sensor

**DOI:** 10.3390/s26051657

**Published:** 2026-03-05

**Authors:** Harnoordeep Singh Mann, Hitesh Bhogilal Vasava, Hamid Mohebzadeh, Mojtaba Naeimi, Naoya Kadota, Manjeet Singh, Prasad Daggupati, Asim Biswas

**Affiliations:** 1 School of Environmental Sciences, University of Guelph, Guelph, ON N1G 2W1, Canada; harnoord@uoguelph.ca (H.S.M.); hvasava@uoguelph.ca (H.B.V.); mnaeimi@uoguelph.ca (M.N.); nkadota@uoguelph.ca (N.K.); 2 School of Engineering, University of Guelph, 50 Stone Rd E, Guelph, ON N1G 2W1, Canada; hmohebza@uoguelph.ca (H.M.); pdaggupa@uoguelph.ca (P.D.); 3 College of Agricultural Engineering & Technology, Punjab Agricultural University, Ludhiana 141004, Punjab, India; deancoaet@pau.edu

**Keywords:** ephemeral gully erosion, soil conservation, image processing techniques, precision agriculture, gully morphology measurement, soil erosion monitoring

## Abstract

**Highlights:**

**What are the implications of the main findings?**

- Novel Pin-artboard tool measures ephemeral gully cross-sections with <1% error
- Automated image processing reduces field time by 90% vs. conventional methods
- Cost-effective alternative to LiDAR at 1–2% equipment cost, comparable accuracy
- Validated across irregular profiles and two Ontario field sites
- Enables event-based monitoring for precision agriculture applications

**Abstract:**

Soil erosion, particularly ephemeral gully (EG) erosion, poses a significant threat to agricultural sustainability and ecosystem health. Despite their substantial impact on soil degradation, EGs have been relatively understudied, primarily due to their temporary nature and the limitations of existing measurement techniques. This study introduces an integrated approach for quantifying and analyzing EGs, addressing the critical need for accurate and scalable measurement methods. Our methodology combines three key components: (1) an updated portable field tool (Gulliometer), which improves upon existing designs to enhance data collection in diverse field conditions; (2) a standardized image acquisition protocol that ensures consistent, high-quality data capture; and (3) an image processing technique leveraging easy repetitive analysis of gully cross-sections. Laboratory validation using known geometric shapes demonstrated the high precision of our methodology, with error rates below 1%. Field applications in two distinct locations in Ontario, Canada, further confirmed the practicality and effectiveness of our approach under varied environmental conditions. This approach not only advances our understanding of ephemeral gully erosion but also aids in the development of effective soil conservation strategies and informed decision-making in land management.

## 1. Introduction

Soil erosion remains a critical environmental challenge in the 21st century, with profound implications for agricultural sustainability and ecosystem health [1]. Among the various forms of soil erosion, ephemeral gully (EG) erosion presents unique challenges due to its dynamic nature and significant contribution to soil loss [2,3]. Despite their modest dimensions (typically 0.5–1.5 m width, 0.3–0.8 m depth) compared to permanent gullies, ephemeral gullies are particularly critical in precision agriculture contexts. EGs account for 20–60% of total sediment yield from agricultural fields [4], with individual events potentially removing 50–150 tons of soil per kilometer of channel [5], creating significant within-field heterogeneity in soil depth, organic matter content, and nutrient availability that directly impacts crop productivity and input efficiency [6]. Unlike permanent gullies, which are typically excluded from cultivated areas, EGs occur within actively managed field zones, directly interfering with precision agriculture operations such as variable rate application equipment, automated guidance systems, yield monitors, and planting equipment [6]. Their rapid development during intense rainfall events (hours to days) and seasonal obliteration through tillage make monitoring particularly challenging, as traditional annual soil surveys miss inter-survey erosion events, and effective conservation practice placement requires event-based, high-frequency measurement [7]. Furthermore, EG development is controlled by complex interactions between topography, soil properties, crop management, and rainfall characteristics [5], requiring high-resolution validation data for erosion model calibration and BMP optimization that existing measurement approaches cannot cost-effectively provide [8].

The transient nature of EGs, coupled with their high soil loss rates, makes their quantification particularly problematic, as conventional soil loss equations, such as the Universal Soil Loss Equation (USLE), fail to account for this type of erosion [9]. The accurate measurement and analysis of EGs have long been hindered by limitations in existing methodologies. While remote sensing techniques such as LiDAR and aerial imagery have advanced gully erosion measurement [10,11], they are often constrained by high costs, data resolution issues, and accuracy limitations influenced by field conditions and stem and canopy density [12]. Moreover, these methods may not effectively capture the full dynamics of EGs under varying field conditions [13].

Recent methodological developments in ephemeral gully measurement have introduced several advanced approaches, each with distinct advantages and limitations. Photogrammetric techniques using Structure-from-Motion (SfM) have gained prominence for their ability to create high-resolution 3D models of gully systems [14,15]. Studies by [16,17] demonstrated SfM accuracy within 2–3 cm for gully volume estimation, though these methods require significant post-processing time (several hours per site) and optimal lighting conditions. Terrestrial laser scanning (TLS) offers exceptional spatial resolution (millimeter-scale) and has been successfully applied to permanent gully monitoring [18,19], but the high equipment cost ($50,000–$150,000) and transportation challenges limit its applicability for ephemeral gullies in actively farmed fields. UAV-based photogrammetry has emerged as a promising tool for landscape-scale gully mapping [16], achieving vertical accuracies of 3–5 cm [20]; however, it struggles with narrow ephemeral gullies (<0.5 m width) obscured by crop canopy and requires favorable weather conditions and regulatory compliance [21]. Ground-penetrating radar has been explored for subsurface gully characterization [22], but interpretation complexity and equipment costs remain barriers. Traditional pin-frame profilers have shown promise in measuring cross-sectional gully areas within mature crop canopies [13], though manual data recording introduces operator variability and time constraints. Refs. [12,23] considered volume calculated with micro-topographic profile meters as the most precise method, using it as a reference for comparison to other techniques. However, existing tools are often limited by their mobility, the size of gullies they can measure, and the manual or semi-automated nature of data processing, which can be time-consuming and prone to human error.

A comparison of various soil profilers, based on their structure, mobility, and other properties, is presented in Table 1. These profilers typically utilize either manual recording of cross-sectional data on graph paper or image capture with a background panel marked with lines at regular intervals. The subsequent digitization process, which involves inputting (x, y) coordinates into a computer, is time-consuming and limits the profilers’ repetitive use for extensive data collection.

Despite these technological advances, a critical gap remains in the availability of cost-effective, field-portable tools specifically designed for rapid, accurate cross-sectional measurement of ephemeral gullies in agricultural settings [24]. Recent comparative studies [25,26] indicate that while high-end remote sensing approaches provide excellent spatial coverage, they often lack the temporal resolution needed for event-based ephemeral gully monitoring, particularly during and immediately after erosive rainfall events when field access with heavy equipment is impractical. Furthermore, the trade-off between measurement accuracy, equipment cost, portability, and ease of use creates a persistent methodological challenge for soil conservation practitioners and researchers working at field and farm scales [27].

**Table 1:** Comparison of different soil topographic profilers evaluating structural dimensions, gully width measurement, cross-sectional digitization, and portability.

	Structural Dimensions	Gully Width Measurement	Cross-Sectional Digitization	Portability	Challenges
Microtopographic profiler [28]	1 m long (2 cm spacing)	1 m	Photographed and digitized in lab *	Base foundation is installed for leveling	Foundation installation is required every time for leveling. Can measure up to 1 m, and the digitization requires manual input of (x, y) values
Gulliometer [29]	40 inch (1.01 m) long	1 m	Recorded on graph paper and later digitized in lab	The equipment is installed at the site to manually draw the cross-section on the graph paper	Time-consuming and repetitive readings are not possible
Soil Erosion Bridge [30]	2 m × 2 m (square frame)	Can measure up to 2 m but requires movement of profiler over the square frame	Photographed and digitized in lab *	2 m × 2 m square frame covers 4 m^2^, causing portability issues and difficulty in multiple measurements	Heavy and large equipment covers a large space Up to 2 m but requires movement, and the digitization requires manual input of (x, y) values
Micro-topographic profiler [13]	1 m long (1 cm spacing)	1 m	Photographed and digitized in lab *	Heavy metal frame and base foundation are installed for leveling	Foundation installation is required every time for levelling. Can measure up to 1 m and the digitization requires manual input of (x, y) values

* The device has a panel in the background, which has black horizontal lines 2 cm apart painted on its front face. The photographs are captured, and data analysis is carried out by the computer, giving (x, y) values for each rod.

Given these limitations, there is a critical need to develop a comprehensive methodology that combines an improved field measurement tool with advanced image processing techniques capable of accurately and efficiently measuring ephemeral gully cross-sections. This study addresses this gap by introducing a novel protocol that integrates three key components: an updated portable field tool (Gulliometer) that builds upon and improves existing designs to enhance data collection in diverse field conditions; a standardized image acquisition process that ensures consistent, high-quality data capture; and an innovative image processing technique that leverages artificial intelligence to automate and enhance the accuracy of gully cross-section analysis.

This approach represents a significant advancement over existing methods, offering several key innovations. The improved field data collection through an optimized portable tool design addresses limitations of previous instruments, while the standardized image acquisition protocol minimizes errors and ensures data consistency across measurements. The application of advanced image processing and AI techniques automates the analysis of gully cross-sections, dramatically reducing processing time and human error while increasing measurement precision. By integrating these components into a cohesive methodology, we enable more frequent, accurate, and comprehensive monitoring of ephemeral gully dynamics. This system not only enhances the accuracy and efficiency of field measurements but also opens new avenues for data analysis and interpretation in soil erosion research.

The primary objective of this study is to develop and validate this comprehensive framework for measuring EG channels in field conditions. The present study substantially expands upon preliminary findings reported previously [31]. We aim to develop and test an updated portable tool (Gulliometer) for measuring EGs in the field, improving upon the capabilities of existing tools. Additionally, we seek to establish a standardized protocol for image acquisition to ensure consistent, high-quality data collection. A key focus is on employing advanced image-based techniques and AI algorithms to measure and analyze cross-sectional EG channels, offering a novel approach to understanding soil erosion processes. Finally, we validate the accuracy and efficiency of this integrated methodology through laboratory tests and field applications.

This research not only addresses the technical challenges of EG measurement but also contributes to the broader field of precision agriculture and soil conservation. By providing a more accurate, efficient, and scalable method for monitoring EG erosion, this study aims to enhance our understanding of soil erosion dynamics and inform the development of more effective soil conservation strategies. This methodology also addresses key themes in sensor technology and environmental monitoring relevant to the *Sensors* journal’s scope. The Pin-artboard represents a self-calibrating optical measurement system that achieves sub-centimeter accuracy using an integrated physical reference standard (37 mm pin head diameter), eliminating dependency on external scaling objects that can introduce positioning errors or calibration inconsistencies. The automated image processing demonstrates robust computer vision algorithms for feature extraction (pinhead detection, boundary identification, and position calculation) under variable field conditions, including diverse soil backgrounds, lighting variations, and surface textures. The approach exemplifies cost-effective precision sensing design, achieving measurement accuracy comparable to professional surveying equipment at approximately 5% of the capital cost through innovative integration of simple, portable hardware with advanced image analysis algorithms. These contributions advance the state of the art in field-deployable environmental sensing systems, with methodology applicable beyond soil erosion to any application requiring rapid, accurate cross-sectional profiling of surface features (e.g., infrastructure monitoring, geomorphological studies, or agricultural surface characterization).

## 2. Materials and Methods

### 2.1. Design of the Pin-Artboard or Gulliometer

**Figure 1:**
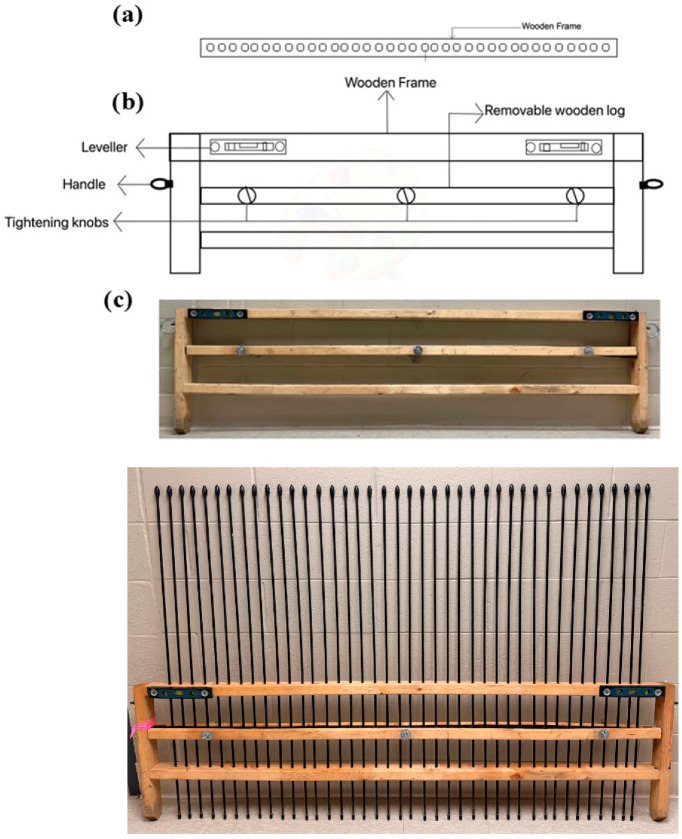
Design of the Pin-artboard with components; (**a**) top-view showing the holes where the removable pins are placed, (**b**) front-view showing the wooden frame with the labeled components, and (**c**) actual developed Pin-artboard with and without the pins placed in.

Ephemeral gullies, characterized by their less extensive dimensions compared to permanent gullies, necessitate specialized measurement tools. The image acquisition Pin-artboard or Gulliometer, designed and developed at the University of Guelph, Canada addresses this need effectively. The Pin-artboard’s wooden structure offers robustness while maintaining portability (easy to lift) for field use. Pins are positioned at equal distances and are easily removable. A leveler is attached to both sides of the frame of the Pin-artboard to ensure the leveling of the frame during each reading. Considering the average width of ephemeral gullies, typically ranging from 0.5 m to 1.50 m [4], the Pin-artboard spans approximately 1.82 m, accommodating 40 pins, each spaced 4 cm apart with 11 cm space left on each side (Figure 1).

### 2.2. Development of Pin-Artboard

The Pin-artboard, measuring 1.82 m × 0.5 m, surpasses previous designs used by [13,32] in terms of gully width coverage. Its design includes circular handles and a leveler for stability and eliminating the installation of a base foundation every time before data collection, which was used by [13] (Figure 1). The 4 cm pin spacing was selected based on a systematic evaluation of the trade-offs between spatial resolution, measurement accuracy, tool portability, and field practicality. The 40-hole configuration with erosion pins covers the size of the EGs easily in a single reading, which has been a limitation in the Gulliometer by [30] and the Microtopographic profiler by [8]. The tool’s design ensures that when placed over a gully, the pins adapt to its shape.

Ephemeral gullies in agricultural fields typically exhibit bottom widths ranging from 20 to 150 cm [4], with relatively smooth, erosionally formed profiles lacking the sharp morphological features found in larger, permanent gullies. Previous studies using 1 cm spacing [13] and 2 cm spacing [33] demonstrated that for gullies with bottom widths >30 cm, the additional detail captured by <2 cm spacing did not significantly improve cross-sectional area estimation accuracy (<2% difference). However, for very narrow channels (<20 cm), finer spacing provides better representation of sharp morphological transitions.

Finer pin spacing introduces several practical challenges: tool weight and portability (1 cm spacing would require 182 pins vs. 46 pins at 4 cm spacing, increasing tool weight from 8 kg to approximately 18 kg); field deployment time increases proportionally with pin density; image processing complexity increases with higher pin density; closely spaced pins are more susceptible to mutual interference and bending when inserted into compacted or stony soils.

Laboratory validation with varying pin spacing (2 cm, 4 cm, 6 cm) on simulated irregular profiles demonstrated that 4 cm spacing achieved <1% area estimation error for profiles with characteristic wavelengths >12 cm (representing 3× the pin spacing, consistent with the Nyquist sampling criterion). Field measurements of 45 ephemeral gullies in Ontario agricultural fields revealed that 94% had characteristic morphological features >15 cm, making 4 cm spacing adequate for accurate representation.

### 2.3. Image Acquisition and Processing

Precise image acquisition is critical for accurate analysis. Images were captured using a smartphone camera positioned approximately 2–2.5 m perpendicular to the Pin-artboard face. The smartphone’s built-in grid overlay feature was used to ensure alignment and minimize angular distortion. Care was taken to ensure the levelers visible in the image appeared horizontal before capturing each image.

**Figure 2:**
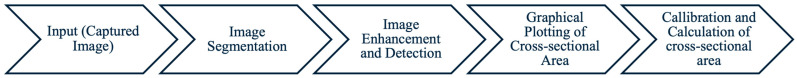
Flow chart of the procedure for image processing.

**Figure 3:**
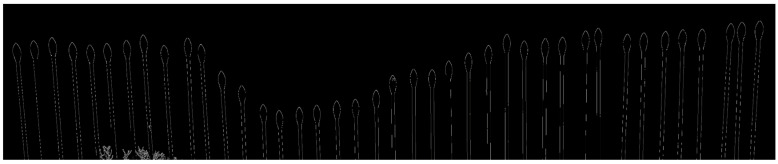
Snapshot of the image after the segmentation and Canny edge detection.

**Figure 4:**
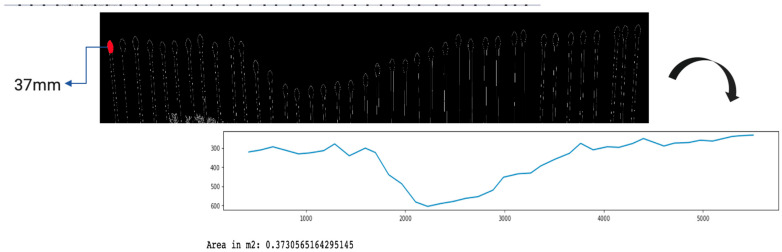
Snapshot of the final output after the image processing.

The image processing workflow (Figure 2) consisted of five main steps:

(1) Image Segmentation: Each captured image was cropped to isolate the target area containing the pins and gully profile, removing extraneous background elements (Figure 3).
(2) Image Enhancement: Canny edge detection [32] was applied to the segmented image to identify pin boundaries against the black background (Figure 4). This algorithm detects edges based on intensity gradients, making it well-suited for identifying the distinct pinheads against the contrasting background.
(3) Pin Detection: The edge-detected image was processed to identify the topmost point of each pinhead. These points represent the soil surface contact positions along the cross-section.
(4) Profile Reconstruction: The sequence of pin top points was plotted to visualize the gully cross-sectional shape (Figure 4).
(5) Calibration and Area Calculation: The known pinhead diameter (37 mm) served as an internal calibration reference, eliminating the need for external scaling objects. The image was calibrated by measuring the pinhead diameter in pixels and calculating a pixel-to-millimeter conversion factor. Cross-sectional area (initially in pixels^2^) was then converted to square meters using this calibration factor. The area under the profile curve was computed using numerical integration.

All processing was implemented in Python (version 3.9.18) using the Jupyter Notebook environment (version 6.5.4), utilizing standard image processing libraries. Processing time per image was approximately 10–15 s on standard laptop hardware, representing a significant time savings compared to manual digitization methods that require manual input of (x, y) coordinates for each pin.

**Figure 5:**
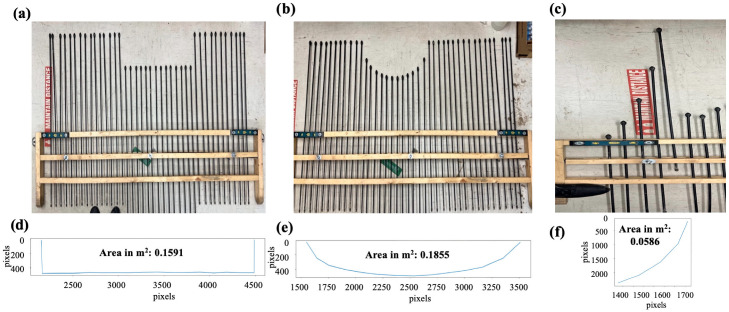
Simulated shapes on the Pin-Artboard in a laboratory setting: (**a**) rectangular, (**b**) semi-circular, and (**c**) following the function y = x^2^. The digitized graphical images of these simulated shapes on the Pin-Artboard, along with estimated areas using image-processing techniques, are presented in (**d**) rectangular, (**e**) semi-circular, and (**f**) following the function y = x^2^.

To test the accuracy, this image processing technique is then tested in a laboratory setting. The pins of the Pin-artboard are set up in the shapes of a semicircle, a rectangle, and a parabolic function. Preliminary testing with geometric shapes (Figure 5a–c) validated the tool’s accuracy in replicating and estimating areas of shapes.

### 2.4. Field Measurements

**Figure 6:**
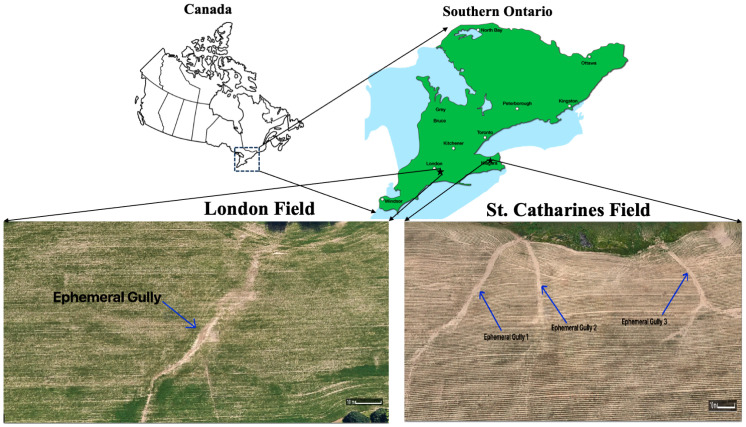
Locations of the fields in Southern Ontario for the field testing of the Pin-artboard in London and St. Catharines, Ontario, Canada.

Field tests were conducted in London and St. Catharines, Ontario, Canada (Figure 6). The London site (42°53′36.7″ N 81°07′55.0″ W) features a primary ephemeral gully with subsidiary channels, while the St. Catharines location (43°08′01.9″ N 79°17′30.4″ W) hosts multiple distinct ephemeral gullies. These tests provided valuable data on the tool’s effectiveness in diverse field conditions. For the field measurements, the subsequent steps involve placing the Pin-artboard or Gulliometer on the ephemeral gully, ensuring the leveling using the levelers, letting the pins slide, and taking the cross-sectional shape of EG. After that, the nuts are tightened to lock the position of the pins, and an image is captured using a digital camera from the front, ensuring the alignment of the image.

## 3. Results

### 3.1. Validation of the Cross-Sectional Area

To validate our method, we compared the calculated areas against actual areas of known shapes. The results, exhibiting marginal error, affirm the reliability of our approach for ephemeral gully volume estimation (Table 2). This validation is crucial; the actual area of the shapes is compared with the area calculated using image-processing techniques. The dimensions of the rectangular shape are 22.8 × 69.5 cm, and the radius of the semicircle is 10.875 cm. The percentage error is defined as:

$$Percentage\;Error=\left|\frac{v_{A\;}-\;v_{E}}{v_{E}}\right|\times 100\%$$

$$v_{A}=estimated\;value\mathrm{and}v_{E}=exact\;value\;\text{further corroborates the method’s precision.}$$

The low error percentages attest to the Pin-artboard’s efficacy in estimating the cross-sectional area. Profilers used by [13,33] have 1 cm and 2 cm spacing between the pins, respectively. The Pin-artboard has 4 cm spacing that provides accurate results with a low error percentage. This finding is critical for field applications, demonstrating its versatility and reliability across various scenarios. The potential of image processing techniques in gully analysis is thus highlighted, with the Pin-artboard playing a crucial role in advancing our understanding of these environmental features.

### 3.2. Field Application

**Figure 7:**
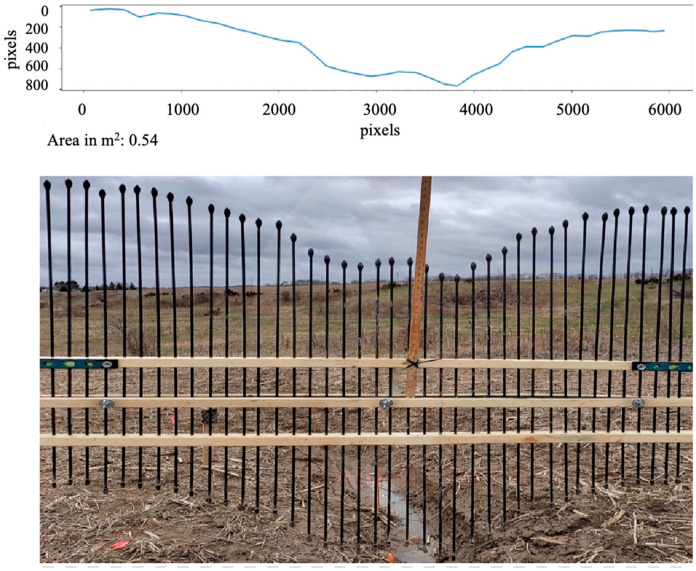
Digitized graphical image with estimated area for EG cross-section in the London field.

**Figure 8:**
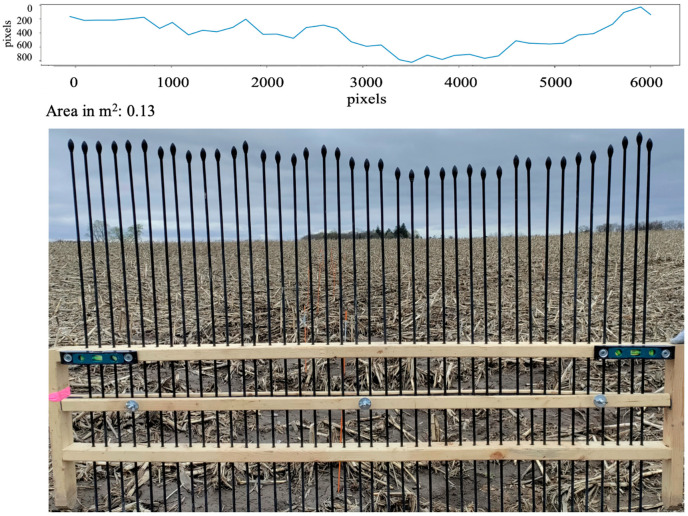
Digitized graphical image with estimated area for EG cross-section in the St. Catharines field.

Upon successful laboratory validation, the Pin-artboard’s application extended to field conditions, measuring ephemeral gullies’ cross-sectional shapes. Before the placement of the Pin-artboard on the EG, a very thin metal Pin is placed at the center of the EG, and then the Pin-artboard is placed such that the center of the Pin-artboard aligns with the Pin placed in the EG. The edge of the EG is considered to be the side pins of the Pin-artboard. Figure 7 and Figure 8 show the Pin-artboard with the graph of the depth of EG at a location from the London and St. Catharines field, respectively.

### 3.3. Field Measurement Summary

The Pin-artboard was successfully deployed at both field sites. At the London site, measurements were conducted along the primary ephemeral gully and subsidiary channels. At the St. Catharines location, measurements captured multiple distinct ephemeral gully cross-sections. Two representative examples are presented in Figure 7 and Figure 8, showing cross-sectional areas of 0.54 m^2^ (London) and 0.13 m^2^ (St. Catharines), illustrating the range of gully morphologies encountered. A limitation of the current study is the absence of comprehensive statistical analysis across multiple cross-sections, repeatability testing with replicate measurements at the same location, or direct comparison with established reference methods (e.g., total station surveys) in the field. Such quantitative validation would strengthen confidence in the methodology’s performance across the full range of field conditions, environmental variability, and operator differences. The field applications presented here demonstrate proof-of-concept functionality but represent preliminary deployment rather than exhaustive validation.

### 3.4. Challenges in Field Measurements

While the Pin-artboard’s design optimizes mobility and ease of use, field measurements present unique challenges. The accuracy of area calculations is contingent on the photograph’s angle. Optimal accuracy is achieved when images are captured perpendicularly to the Pin-artboard. Angled photographs can skew pixel calibration, affecting area calculations. To overcome this challenge, we used the grid lines feature in the smartphone camera to make sure that the camera was not tilted while capturing the image. This in-built feature of a smartphone helped to get rid of an additional structure to hold a camera at a straight angle, as used by [33]. The tool and methodology’s benefits in field conditions are substantial, offering a practical tool for repetitive measurements and enhancing our understanding of ephemeral gully morphology and dynamics.

## 4. Discussion

### 4.1. Performance and Methodological Advantages

The Pin-artboard methodology achieved error rates below 1% in laboratory validation with known geometric shapes (Table 2), demonstrating measurement precision under controlled conditions. This performance is comparable to micro-topographic profilers, which [33] considered the most precise field method for gully cross-sectional measurement.

Compared to existing profilers (Table 1), the Pin-artboard offers several practical advantages. The Gulliometer developed by Smith [30] required manual recording on graph paper, making repetitive measurements time-consuming. The Soil Erosion Bridge [31] covers a 2 m × 2 m area, creating portability challenges. Previous profilers [8,11,29] required a base foundation installation for leveling at each location. In contrast, the Pin-artboard incorporates integrated levelers, eliminating base foundation installation and reducing setup time to approximately 5–10 min per measurement.

The most significant advancement is the elimination of manual digitization. Traditional methods required either manual drawing on graph paper [30] or manual input of (x, y) coordinates from photographs [8,11,13,31], processes taking 15–30 min per cross-section and subject to individual interpretation variability. The automated image processing extracts pin positions directly from images in 10–15 s with pixel-level precision, eliminating subjective interpretation and enabling higher throughput for studies requiring many measurements.

The equipment cost of approximately $300–350 (plywood $40–60, aluminum pins $80–100, bubble levels $20–30, hardware $15–25, finishing $10–20) represents a small fraction of total station costs ($5000–15,000), terrestrial laser scanners ($50,000–150,000), or UAV systems ($3000–8000). While advanced remote sensing technologies offer high accuracy and spatial coverage [14,15,16,17,18,19,20,21], they require substantial capital investment and may need specialized expertise. Recent studies demonstrate Structure-from-Motion accuracy of 2–3 cm [14,15] but require several hours of post-processing per site. Terrestrial laser scanning achieves millimeter-scale resolution [18,19] but faces equipment transportation challenges. UAV-based approaches provide landscape coverage [20,21] but struggle with narrow gullies obscured by crop canopy. As noted by [24,25], the persistent challenge is balancing measurement accuracy with equipment cost, portability, and ease of use. The Pin-artboard addresses this gap by providing adequate accuracy for most monitoring applications at a cost enabling frequent, extensive measurement programs.

### 4.2. Critical Limitations

Several important limitations must be acknowledged:

Validation scope: Laboratory testing employed only regular geometric shapes (rectangle, semicircle, and parabola). Real ephemeral gullies exhibit irregular, asymmetric profiles with undercuts, bank collapse features, and surface roughness. The <1% error achieved with regular shapes may not fully represent performance on highly irregular natural profiles. Future validation should include: (1) comparison with total station or TLS measurements at multiple field locations; (2) repeatability testing with replicate measurements by different operators; (3) systematic testing across varying gully widths, depths, and morphological complexity.

Field validation intensity: The current field application presented only two representative cross-sections (Figure 7 and Figure 8), demonstrating proof-of-concept functionality rather than comprehensive validation. We did not conduct repeatability assessment, comparison with reference methods at the same locations, or testing across diverse conditions. This represents a preliminary deployment requiring expanded validation before the methodology can be considered fully validated for operational use.

Photographic angle sensitivity: Measurement accuracy depends on capturing images perpendicular to the Pin-artboard face. While the smartphone grid overlay aids alignment, this relies on operator judgment. We did not quantify angular error effects on calculated areas. Angled photographs can introduce systematic errors in pixel calibration. Future development should include a simple mounting bracket ensuring consistent perpendicular positioning, particularly for inexperienced operators.

Environmental factors: We did not systematically evaluate lighting effects, though field experience suggested diffuse lighting (overcast) provided more consistent results than direct sunlight with shadows. Canny edge detection is inherently sensitive to lighting conditions [32], which can affect pinhead boundary detection. Heavy crop residue prevented pins from reaching the true soil surface in some locations, requiring gentle removal, but we did not quantify the frequency or magnitude of this interference. Pin deflection in hard or stony soils was observed but not systematically documented. Users should prioritize favorable conditions (overcast lighting, minimal residue, moderate soil moisture) when possible.

Width and spacing constraints: The 1.82 m span accommodates typical ephemeral gully widths (0.5–1.5 m) [4,18] but cannot capture wider features in a single measurement. The 4 cm pin spacing may provide reduced accuracy for very narrow gullies (<20 cm width) with sharp morphological transitions, though the majority of agricultural ephemeral gullies exceed this width based on field observations and literature [4].

### 4.3. Applications in Precision Agriculture

Despite acknowledged limitations, the methodology supports several important applications:

Event-based monitoring: The tool’s portability enables measurements during and immediately after erosive rainfall events, when heavy surveying equipment cannot access muddy fields. This timing captures ephemeral gullies before modification by tillage or natural processes, addressing the limitation identified by [7] that traditional annual surveys miss inter-survey erosion events.

Best management practice evaluation: Quantifying cross-sectional changes over time supports evidence-based assessment of conservation practice effectiveness. Specific applications include: (1) grassed waterway design-precise geometry enables hydraulic calculations for optimal sizing, with repeated measurements documenting performance and maintenance needs; (2) controlled traffic farming-identifying preferential flow paths enables adjustment of traffic lane positioning to minimize concentrated flow erosion [6]; (3) variable tillage zoning-sub-field erosion mapping guides precision tillage intensity decisions; (4) cover crop assessment-event-based monitoring quantifies impacts on gully initiation and expansion; (5) conservation practice validation-before-after comparisons and multi-year monitoring document BMP performance over time, as emphasized by [8].

Research scalability: The ability to conduct 20–40 measurements per field day enables larger sample sizes for spatial variability analysis and treatment comparisons in controlled experiments. The low equipment cost expands research capacity for graduate students, educational institutions, extension programs, and developing country applications where expensive surveying equipment is unavailable [27].

### 4.4. Future Development

Several priorities would enhance the methodology:

(1) Comprehensive field validation: Direct comparison with total station measurements across ≥20 field locations spanning diverse morphologies, soil types, and conditions. Repeatability testing with multiple operators to quantify precision and inter-operator variability.
(2) Camera mounting system: Development of a simple bracket ensuring consistent perpendicular positioning would eliminate operator-dependent variation, particularly beneficial for inexperienced users.
(3) Image quality assessment: Automated detection of problematic images (excessive shadows, poor focus, inadequate contrast) with immediate feedback would enable re-acquisition before leaving the field site.
(4) User interface improvements: A smartphone application enabling field-deployed processing, immediate result visualization, GPS-tagged data management, and quality control feedback would streamline workflows for large-scale monitoring.
(5) Erosion model integration: Standardized data formats for incorporating measurements into watershed models (WEPP, SWAT, and AnnAGNPS) would enhance ephemeral gully representation in predictive modeling.
(6) Multi-institutional testing: Collaborative validation across research groups and geographic regions would assess methodology transferability and identify context-specific challenges.

The Pin-artboard methodology represents a practical advancement in accessible ephemeral gully monitoring, balancing accuracy, cost, and field practicality. While important limitations require future attention, the demonstrated capabilities support expanded applications in precision agriculture, conservation practice evaluation, and erosion research at scales previously constrained by equipment costs and deployment logistics.

## 5. Conclusions

The Pin-artboard methodology provides an accurate (<1% error in laboratory validation), portable, and cost-effective (~$300–$350 equipment investment) approach for measuring ephemeral gully cross-sectional morphology. The integration of simple field instrumentation with automated image processing addresses critical limitations of existing profilers, including time-consuming manual data recording, base foundation installation requirements, and subjective manual digitization of measurements.

This methodology directly supports evidence-based precision agriculture and soil conservation decision-making through several specific applications:

(1) Grassed waterway design and performance monitoring: Precise cross-sectional geometry enables hydraulic calculations for optimal waterway sizing. Repeated measurements document sediment deposition patterns, identify maintenance needs (vegetation re-establishment, sediment removal), and quantify effectiveness in reducing ephemeral gully erosion.
(2) Controlled traffic farming optimization: High-frequency monitoring identifies preferential flow paths and incipient gully formation areas, enabling adjustment of permanent traffic lane positioning to minimize concentrated flow erosion.
(3) Variable tillage intensity zoning: Sub-field mapping of erosion vulnerability guides precision tillage management, concentrating conservation tillage in high-risk zones while using conventional tillage where appropriate.
(4) Cover crop effectiveness assessment: Event-based monitoring quantifies cover crop impacts on gully initiation and expansion, supporting evidence-based decisions on species selection, seeding rates, and deployment strategies.
(5) Conservation practice validation: Before-after comparisons and multi-year monitoring document BMP performance, providing accountability for conservation investments and identifying practice modifications to improve effectiveness.

Beyond immediate management applications, the methodology enables research on ephemeral gully formation processes, temporal dynamics, and controlling factors that would be impractical with expensive surveying equipment. The cost accessibility expands research capacity for graduate students, educational institutions, extension programs, and developing country applications. Important limitations requiring future attention include: (1) validation limited to regular geometric shapes rather than irregular natural profiles; (2) absence of systematic comparison with reference methods (total station, TLS) across multiple field sites; (3) lack of quantified repeatability assessment; (4) uncharacterized effects of lighting conditions, vegetation interference, and pin deflection on measurement accuracy; and (5) operator-dependent camera positioning variability. Addressing these limitations through comprehensive field validation and methodological refinements will strengthen confidence in the Pin-artboard’s performance across the full range of operational conditions.

The methodology represents a significant step toward accessible, practical ephemeral gully monitoring for precision agriculture and conservation applications, with continued development promising further improvements in usability, robustness, and accuracy.

## Figures and Tables

**Table 2:** Comparison between the actual area and the area calculated by image processing.

Shape		Area Using Image-Processing (m^2^)	Actual Area (m^2^)	Error (%)
Regular	Rectangular	0.1590	0.1584	0.30
	Semi-circular	0.1855	0.1856	0.05
Parabolic	f(x): y = x^2^	0.0586	0.0581	0.85

## Data Availability

The datasets presented in this article are not readily available because the data are part of an ongoing study.
